# Overview of Psychiatric Conditions in Adults with Intellectual and Developmental Disabilities (IDD)

**DOI:** 10.1192/j.eurpsy.2025.1553

**Published:** 2025-08-26

**Authors:** H. M. Nguyen, D. Valle, E. Jetter, A. Gomes, A. Lertkitcharoenpo, M. Liu, B. Carr

**Affiliations:** 1 University of Florida College of Medicine, Gainesville, United States; 2 Psychiatry, University of Florida College of Medicine, Gainesville, United States

## Abstract

**Introduction:**

Adults with intellectual and developmental disabilities (IDD) are more likely than the general population to experience psychiatric disorders, with prevalence rates estimated at 33-40%. These individuals often display atypical symptoms, complicating diagnosis. Primary care providers are often the first to encounter these patients but face challenges due to limited training and “diagnostic overshadowing”—the misattribution of psychiatric symptoms to the IDD itself rather than recognizing comorbid mental health conditions.

**Objectives:**

This study aims to: (1) highlight the prevalence and unique presentations of psychiatric disorders in adults with IDD, (2) discuss diagnostic challenges, particularly diagnostic overshadowing, in primary care, and (3) advocate for collaborative care models to improve diagnostic accuracy and outcomes.

**Methods:**

A literature review across PubMed, Medline, and PsycINFO focused on studies addressing psychiatric prevalence in IDD, diagnostic barriers, and the efficacy of collaborative care models in managing complex cases in primary care.

**Results:**

Adults with IDD show high rates of mood disorders, anxiety, ADHD, and psychotic disorders, often presenting atypically. For example, ADHD may show as prolonged attentional and behavioral difficulties, impacting social and functional skills. Anxiety may present as agitation or sensitivity to routine changes, often misinterpreted as behavioral issues. Mood disorders, especially depression, tend to appear as irritability or somatic complaints, which are frequently attributed to the IDD itself. Psychotic disorders are also prevalent, particularly among individuals with certain genetic syndromes, and complicate diagnosis due to overlapping symptoms.

Diagnostic overshadowing significantly impacts accurate psychiatric diagnosis in adults with IDD, as primary care providers often attribute psychiatric symptoms to the disability itself. Limited IDD-specific training in primary care compounds this issue. Collaborative care models, where primary care providers collaborate with mental health specialists familiar with IDD, show promise in addressing these diagnostic and treatment challenges, especially for complex cases.

**Conclusions:**

Effective psychiatric care for adults with IDD requires specialized provider training, comprehensive evaluations sensitive to atypical presentations, and collaborative care models. Addressing diagnostic overshadowing through improved training and integrated care can enhance psychiatric outcomes for this underserved population. Further research, particularly randomized controlled trials, is needed to develop evidence-based guidelines for tailored care in adults with IDD.

**Disclosure of Interest:**

None Declared

